# Supplementary material for: Serum Leucine-Rich Alpha-2 Glycoprotein in Quiescent Crohn’s Disease as a Potential Surrogate Marker for Small-Bowel Ulceration detected by Capsule Endoscopy
Source: J Clin Med. 2022 Apr 29;11(9):2494. doi: 10.3390/jcm11092494 (PMC9101788; doi:10.3390/jcm11092494)
Supplement: Supplementary file 1 [file jcm-11-02494-s001.zip › LRG-SBCE-JCM-suple-Table-revised.1.pdf]

Supplementary Table S1. Comparison of the patients with and without small bowel ulcer (>0.5 cm)

		**Total** *n*=40	**Presence of small-bowel ulcer, ≥0.5 cm** *n*=11	**Absence of small-bowel ulcer, ≥0.5 cm** *n*=29	***p*-value**
	Lewis score	0 (0–192)	339 (135–3503)	0 (0–0)	<0.0001
SBCE score	CECDAI	3 (0–6)	8 (3–12)	3 (0–6)	0.0012
	CDACE	211 (0–420]	730 (310–940)	210 (0–370)	0.0003
Biomarkers/clinical activity:					
Hb, g/dL		13.7 (12.2–14.7)	13.9 (12.3–14.6)	13.7 (12.2–14.8)	0.9396
Plt ×10^4/μL		24.6 (21.1–29.2)	24.9 (23.6–26.5)	23.5 (20.7–29.7)	0.4579
Alb, g/dL		4.4 (4.2–4.7)	4.3 (4–4.7)	4.5 (4.2–4.7)	0.1737
CRP, mg/dL		0.08 (0.05–0.15)	0.09 (0.06–0.18)	0.08 (0.05–0.13)	0.3018
LRG, μg/mL		12.3 (8.9–14.1)	14.1 (12.1–16.5)	11.7 (9.3–13.5)	0.0105
CDAI		67 (29.5–89.8)	85 (29–106)	66 (30–85)	0.1680

The data are median (interquartile range). Alb: albumin, CDAI: Crohn's Disease Activity Index, CDACE: Crohn's Disease Activity in Capsule Endoscopy, CECDAI: Capsule Endoscopy Crohn's Disease Activity Index, CRP: C-reactive protein, Hb: hemoglobin, Ht: hematocrit, IQR; inter quartile range, SBCE: small-bowel capsule endoscopy.

Supplementary Table S2. Comparison of the patients with and without Lewis score ≥350

		**Total** *n*=40	**Presence of Lewis score ≥350** *n*=5	**Absence of Lewis score <350** *n*=35	***p*-value**
	Lewis score	0 (0–192)	3503 (1589–3585)	0 (0–135)	<0.0001
SBCE score	CECDAI	3 (0–6)	12 (6.5–15.5)	3 (0–6)	0.0028
	CDACE	211 (0–420)	831 (312–1042)	210 (0–420)	0.0106
SB Ulcer ≥0.5cm (%)		11 (27.5)	5 (100)	1 (17.1)	0.0007
Biomarkers/clinical activity:					
Hb, g/dL		13.7 (12.2–14.7)	13.1 (11.7–14.4)	13.7 (12.2–14.7)	0.5394
Plt $\times 10^4/\mu L$		24.6 (21.1–29.2)	24.7 (20.7–32)	24.5 (21.1–28.5)	0.5531
Alb, g/dL		4.4 (4.2–4.7)	4.2 (3.9–4.3)	4.5 (4.2–4.7)	0.0318
CRP, mg/dL		0.08 (0.05–0.15)	0.09 (0.07–0.26)	0.08 (0.05–0.15)	0.3892
LRG, µg/mL		12.3 (8.9–14.1)	15.3 (14.4–17.3)	11.7 (9.7–13.6)	0.0030
CDAI		67 (29.5–89.8)	106 (88–125)	65 (28–85)	0.0069

The data are median (interquartile range). Abbreviations are explained in the footnote of Supplementary Table S1.

Supplementary Table S3. Comparison with LRG ≥16 µg/ml as the cutoff

		**Total** *n*=40	**LRG ≥16 µg/ml** *n*=5	**LRG <16 µg/ml** *n*=35	***p*-value**
SBCE score	Lewis score	0 (0–192)	196 (90–2092.5)	0 (0–135)	0.0270
	CECDAI	3 (0–6)	12 (5–15)	3 (0–6)	0.0090
	CDACE	211 (0–420)	730 (371–1042)	210 (0–420)	0.0044
Presence of small bowel ulcer ≥0.5 cm (%)		11 (27.5)	3 (60)	8 (22.9)	0.1171
Lewis score ≥350 (%)		5 (12.5)	2 (40)	3 (8.6)	0.1088
Biomarkers/clinical activity:					
Hb, g/dL		13.7 (12.2–14.7)	14.5 (12.8–14.8)	13.7 (12.1–14.7)	0.4867
Plt $\times 10^4$/µL		24.6 (21.1–29.2)	28 (24.4–30.8)	23.6 (20.7–29.4)	0.1706
Alb, g/dL		4.4 (4.2–4.7)	4.3 (3.9–4.5)	4.4 (4.2–4.7)	0.1732
CRP, mg/dL		0.08 (0.05–0.15)	0.21 (0.07–0.38)	0.08 (0.05–0.14)	0.0927
LRG, µg/mL		12.3 (8.9–14.1)	16.8 (16.5–17.6)	11.7 (9.7–13.6)	0.0004
CDAI		67 (29.5–89.8)	85 (81–104)	65 (28–89)	0.0599

The data are median (interquartile range). Abbreviations are explained in the footnote of Supplementary Table S1.

Supplementary Table S4. Comparison by use of biologics

		**Total** *n*=40	**Biologics (-)** *n*=15	**Biologics (+)** *n*=25	***p*-value**
SBCE score	Lewis score	0 (0–192)	0 (0 – 225)	0 [0 - 135]	0.6071
	CECDAI	3 (0–6)	6 (3 – 10)	3 [0 – 4]	0.0224
	CDACE	211 (0–420)	420 (210 – 730)	210 [0 - 311]	0.0357
Presence of small bowel ulcer ≥0.5 cm (%)		11 (27.5)	4 (26.7)	7 (28)	1.0000
Lewis score ≥350 (%)		5 (12.5)	2 (13.3)	3 (12)	1.0000
Biomarkers/clinical activity:					
Hb, g/dL		13.7 (12.2–14.7)	13.8 (12.1 – 14.7)	13.7 [12.3 – 14.7]	1.0000
Plt $\times 10^4$/μL		24.6 (21.1–29.2)	25 (22.3 – 30.5)	23.6 [20.9 – 28.1]	0.2697
Alb, g/dL		4.4 (4.2–4.7)	4.4 (4.2 – 4.7)	4.3 [4.2 – 4.8]	0.9775
CRP, mg/dL		0.08 (0.05–0.15)	0.1 (0.07 – 0.21)	0.08 [0.05 – 0.14]	0.1572
LRG, μg/mL		12.3 (8.9–14.1)	13.9 (11.7 – 14.4)	10.8 [9.5 – 13.3]	0.0314
CDAI		67 (29.5–89.8)	83 (28 – 106)	66 [30 – 86]	0.2883

Biologics (TNF inhibitor: n=20, Ustekinumab: n=4, Vedolizumab: n=1)

The data are median (interquartile range). Abbreviations are explained in the footnote of Supplementary Table S1.
