# Supplementary figures and images for: Serum Leucine-Rich Alpha-2 Glycoprotein in Quiescent Crohn’s Disease as a Potential Surrogate Marker for Small-Bowel Ulceration detected by Capsule Endoscopy
Source: J Clin Med. 2022 Apr 29;11(9):2494. doi: 10.3390/jcm11092494 (PMC9101788; doi:10.3390/jcm11092494)

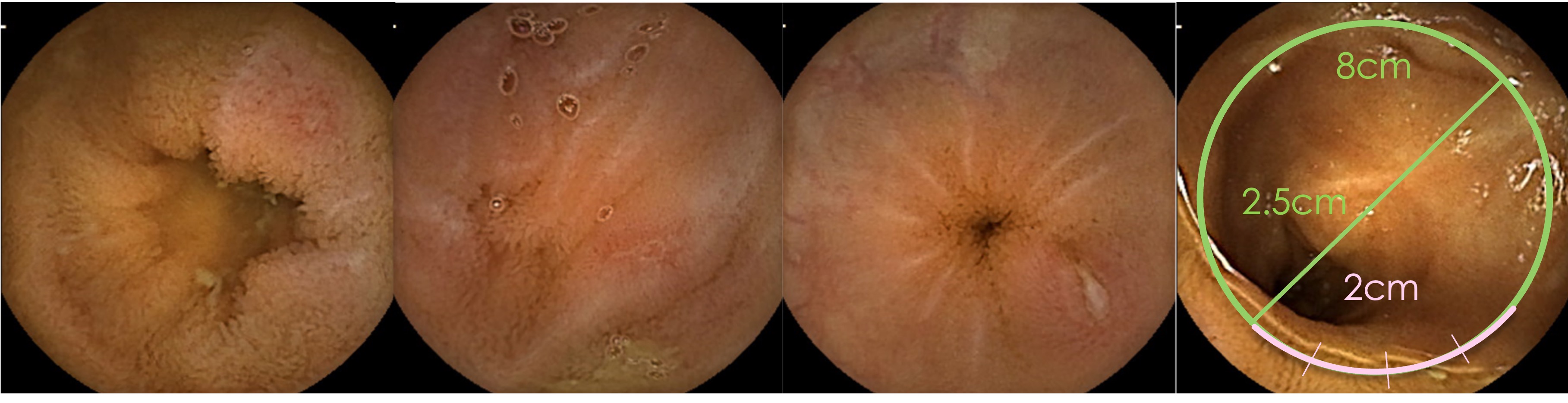

Supplement: Supplementary file 1 [file jcm-11-02494-s001.zip › Supplement-Figure-S1-JCM.jpg]

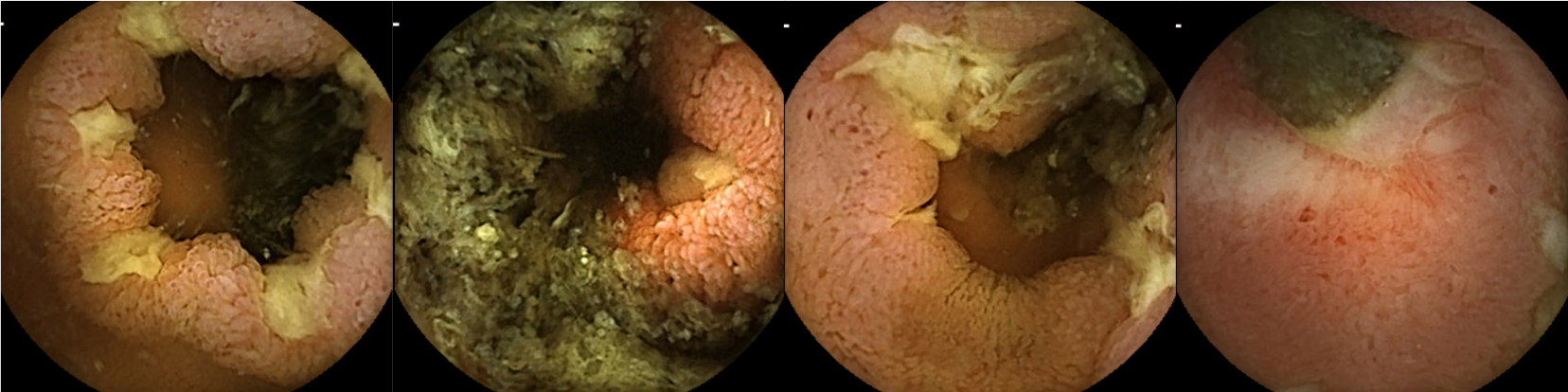

Supplement: Supplementary file 1 [file jcm-11-02494-s001.zip › Supplement-Figure-S2-JCM.jpg]
